# Synthesis and characterization of nanocrystalline diamond from graphite flakes via a cavitation-promoted process

**DOI:** 10.1016/j.heliyon.2019.e01682

**Published:** 2019-05-11

**Authors:** Basma H. Al-Tamimi, Iman I. Jabbar, Haitham M. Al-Tamimi

**Affiliations:** a Department of Materials Engineering, University of Technology, Baghdad, Iraq; b Department of Applied Science, University of Technology, Baghdad, Iraq; c Department of Production Engineering and Metallurgy, University of Technology, Baghdad, Iraq

**Keywords:** Nanotechnology, Materials science

## Abstract

Herein, we describe the multi-step synthesis and characterization of monodisperse cubic-structured nanocrystalline diamond particles, showing that they can be easily prepared from graphite flakes under ambient conditions. The above synthesis features the conversion of graphite flakes into graphene oxide (via a modified Hummer's method) and its subsequent transformation into nanodiamond under the action of ultrasonication-induced cavitation, with the nucleation and growth of nanodiamond particles being strongly influenced by the incorporation of a specific metal oxide spacer material. Overall, the developed method is demonstrated to be superior to conventionally used ones, exhibiting the advantages of simplicity, high yield, and upscaling potential.

## 1 Introduction

Carbon, one of the most interesting and nature-abundant elements, can exist in the form of several allotropes (diamond, graphite, fullerene, carbon nanotubes, graphene, etc.) that differ in the arrangement of carbon atoms within their unit cells [1, 2] and therefore exhibit remarkably variable properties. The recent breakthroughs in the fields of nanoscience and nanotechnology [2, 3] allow the synthesis of nanostructured carbon materials that possessing unique physical and mechanical properties and used in the most critical applications of supramolecular chemistry, mechanical and molecular device fabrication, and mesososcopic physics [2, 4].

The term “nanodiamond” (ND) encompasses a variety of nanoscale diamond-based materials comprising nanotetrahedral networks and regarded as new members of the nanocarbon family [5]. Although ND was discovered several decades ago, it is still of great interest to researchers working in different fields. Importantly, ND is compatible with the human organism and can be functionalized with different biomolecules, being also suitable for use as a reinforcing material to produce attractive composites for industrial applications. Thus, ND is regarded as a very important novel material which is capable of starting a future scientific revolution. The structure of ND features a network of sp^3^ carbons with small amounts of sp^2^ carbons existing primarily at grain boundaries [4]. Compared to other nanocarbon particles, ND ones exhibit unique structural features, e.g., they contain a π-electron network and abundant surface functional groups that allow for surface reconstruction [4]. Although single-crystal ND particles can be produced by high pressure-high temperature synthesis, the use of elevated temperatures (˃2000 K) and pressures (∼10000 atm) together with low yields hinders the practical application of this method and necessitates the search for suitable alternatives. Thus, ND can be efficiently prepared by chemical vapor deposition and detonation techniques, which, however, are not suitable for scalable production [6, 7].

Several studies had been reported attempts to produce diamond from graphite via ultrasonic technique, in 1986 Flynn reported an alternative strategy to generate the required high temperature and high pressure for graphite-to-diamond conversion in metal melt depending on the generated cavitation energy of ultrasonication technique [8]. Other researchers studied the effect of ultrasonication cavitation energy to produce diamond from graphite powder within hexane and ethanol media [9]. In addition there are many theoretical analysis have shown that due to the effect of the surface tension of carbonic nano-materials like graphene or CNTs, diamond nucleation is preferable inside these carbonic nanostructured materials. Some success was achieved with using different catalyst like Ni, Mn and Co. [10].

Herein, we show that ND can be efficiently prepared from natural graphite powder without the use of high temperatures or pressures by a multi-step procedure based on ultrasonication-induced cavitation. Moreover, the insertion of a specific metal oxide as a spacer material is shown to significantly affect the nucleation and growth of ND particles, and the structural modifications at each stage are confirmed by scanning electron microscopy (SEM), X-ray diffraction (XRD), optical microscopy, and Raman spectroscopy analyses.

## 2 Materials and methods

### 2.1 Materials

Graphite (212μm) was purchased from Nanoshel (USA). KMnO_4_ and NaNO_3_ were obtained from BDH Chemicals (UK), and H_2_SO_4_ and H_2_O_2_ were purchased from Sigma-Aldrich.

### 2.2 Synthesis of graphene oxide (GO)

GO was prepared by a modified Hummer's method. Typically, KMnO_4_ was added in enough quantity as small portions to a stirred suspension of graphite in a mixture of H_2_SO_4_ and NaNO_3_ upon cooling in an ice bath, and the obtained mixture was placed in a warm water bath (35 °C) and further stirred for ∼2 h to afford a brown-yellow solution. For complete oxidation, the above solution was placed in a hot water bath (90 °C) and treated with aqueous H_2_O_2_ upon vigorous stirring until the solution color changed to orange-yellow [11].

### 2.3 Synthesis of graphene, graphene nanoscrolls, and ND

Ultrasonication (23KHz) of GO dispersion induced cavitation and resulted in the formation of graphene sheets and other carbon nanoparticles. Specifically, cavitation was caused by alternating high- and low-pressure cycles initiated by the propagation of ultrasound waves through the GO dispersion. During the low-pressure cycle, these waves induced the formation of microscopic voids or bubbles in the medium that reached a certain volume and collapsed during the high-pressure cycle, which created large local increases of temperature (∼5000 K) and pressure (∼2000 atm) under the action of shear forces [12, 13]. All of these factors resulted in the modification and cleavage of GO sheets and impacted their chemistry and morphology to afford reduced GO sheets. On the other hand, the above shear forces induced the scrolling of some of the produced graphene sheets into graphene nanoscrolls. The modified Hummer's method featured the formation of Mn_2_O_7_ [14] that acted as a spacer material and played an important role in the initiation of ND seed formation:

KMnO_4_ + 3 H_2_SO_4_ → K^+^ + MnO_3_^+^ + H_3_O^+^ + 3HSO_4_^–^

MnO_3_^+^ + MnO_4_^–^ → Mn_2_O_7_

**Fig. 1:**
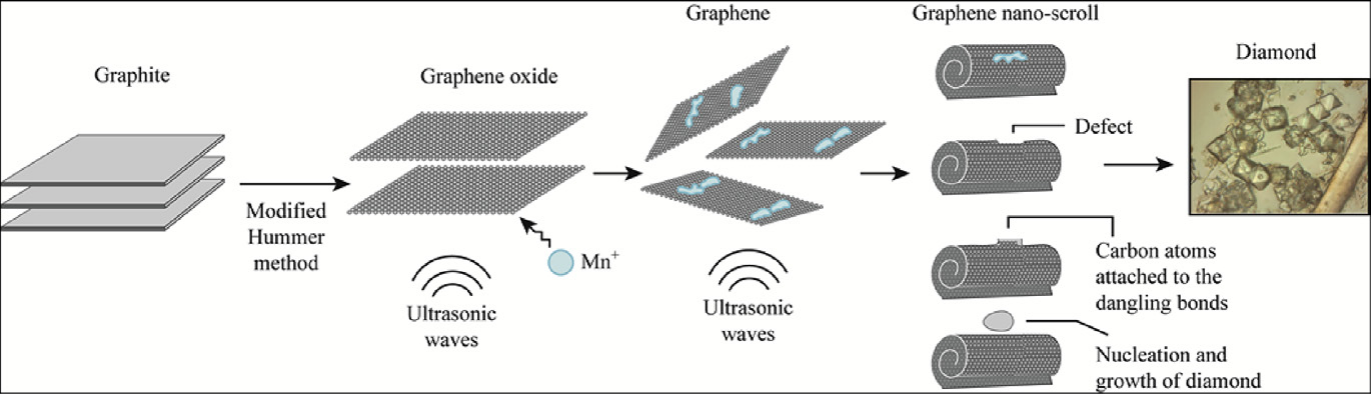
Representation of nucleation and growth of ND.

Carbon nanomaterials, metal oxides, noble metal nanocrystals, polymers, metal-organic frameworks, and even water molecules can also be used as spacer materials [15, 16]. Herein, Mn_2_O_7_ was inserted within the graphene nanoscroll structure via several scrolling steps [12, 13]. Mn_2_O_7_ featured by instability and its tendency to decompose with the release of oxygen, this is primarily occurred at the tips and on the side walls of tubular graphene nanoscrolls. As demonstrated by numerous studies, ND nucleation on side walls may proceed through the formation of diamond nuclei at subsurface damage sites within these walls produced by the decomposition of Mn_2_O_7_ [5]. Subsequently, damaged graphene nanoscrolls having a vacancy defects underwent self-healing, which resulted in the formation of basic ND seeds in which carbon atoms attached to dangling bonds. Finally, the gradual growth and warping up of these nuclei to cluster-shapes regarded as ND seeds afforded crystalline diamond particles as illustrated in Fig. 1 [17,18].

## 3 Results and discussion

### 3.1 Characterization of GO

**Fig. 2:**
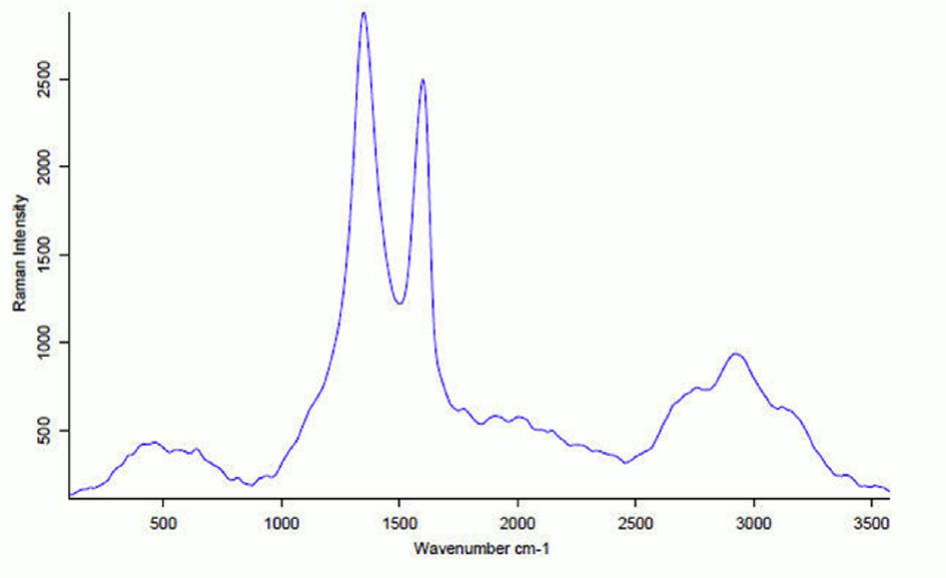
Raman spectrum of Graphene oxide.

Structural information for carbon-based materials can be obtained by using Raman spectroscopy which is a non-destructive widely used characterization technique. G and D bands and their overtones regarded the main features in the Raman spectra of graphitic carbon-based materials. Fig. 2 shows Raman spectra of the prepared GO. From this figure we can notice the presence of the first order G and D peaks at 1590 cm^−1^ and at 1350 cm^−1^ respectively, these two peaks arising from the vibration of sp^2^ carbon atoms. The G peak corresponds to the optical E 2g phonons at the Brillouin zone center resulting from bond stretching of sp ^2^ carbon pairs in both, rings and chains. D peak represents the breathing mode of aromatic rings arising due to the defect in the sample [19]. The intensity of D peak refers to the disorder degree. Also, we can notice the structural disorder due to the presence of weak and broad 2D peak around 2690 cm^−1^. 2D peak is the shift and shape of the overtone of the D peak. 2D peak is attributed to the double resonance transition resulting in the production of two phonons with opposite momentum. A defect activated peak which is called G + D is also readily visible around 2950 cm^-1^.

**Fig. 3:**
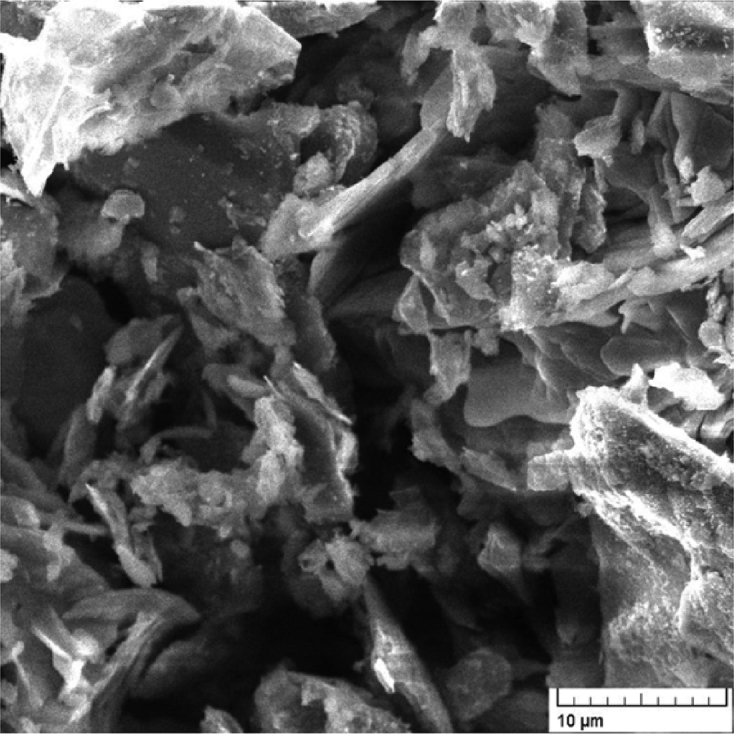
SEM image of Graphene oxide.

The surface morphology of as-prepared GO was investigated by SEM imaging (Fig. 3), which revealed the presence of plate-like structures with irregular edges.

### 3.2 Characterization of graphene sheets (GS)

**Fig. 4:**
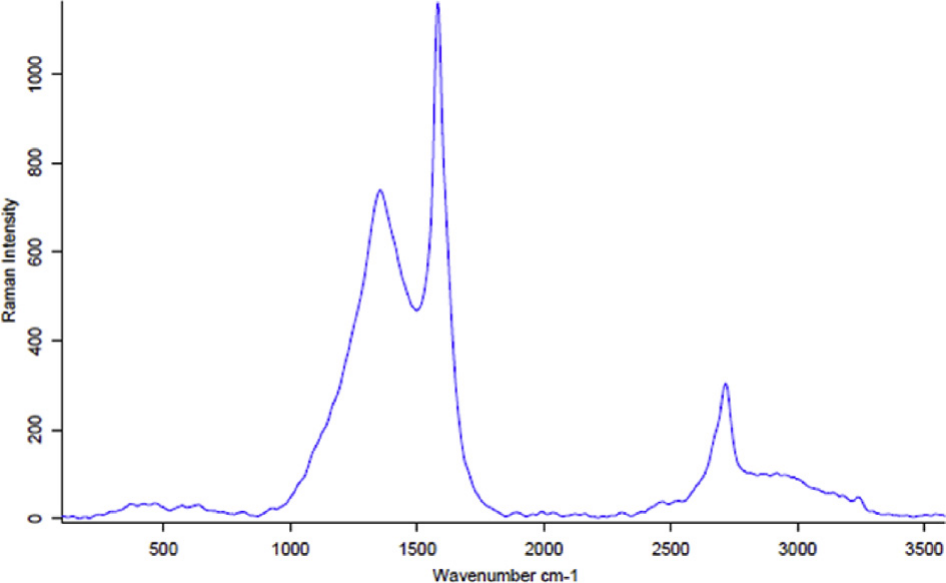
Raman spectrum of Graphene.

Fig. 4 shows the Raman spectrum of graphene produced from GO by ultrasonication, In this figure we can notice the presence of sharp G band around 1585 cm^−1^. This band is an in-plane vibrational mode involving the sp^2^ hybridized carbon atoms that comprises the graphene sheets. The disorder band or defect band (D band) is presence at 1355 cm^−1^. The intensity of the D band is directly proportional to the level of defects in the sample. 2D band is appears at 2690 cm^−1^. 2D band is always strong band in graphene spectra and it is usually used to determine thickness of graphene layers, in this figure we can notice the 2D band is split into several overlapping mode which arises from symmetry lowering that takes place when increasing the number of graphene layers.

**Fig. 5:**
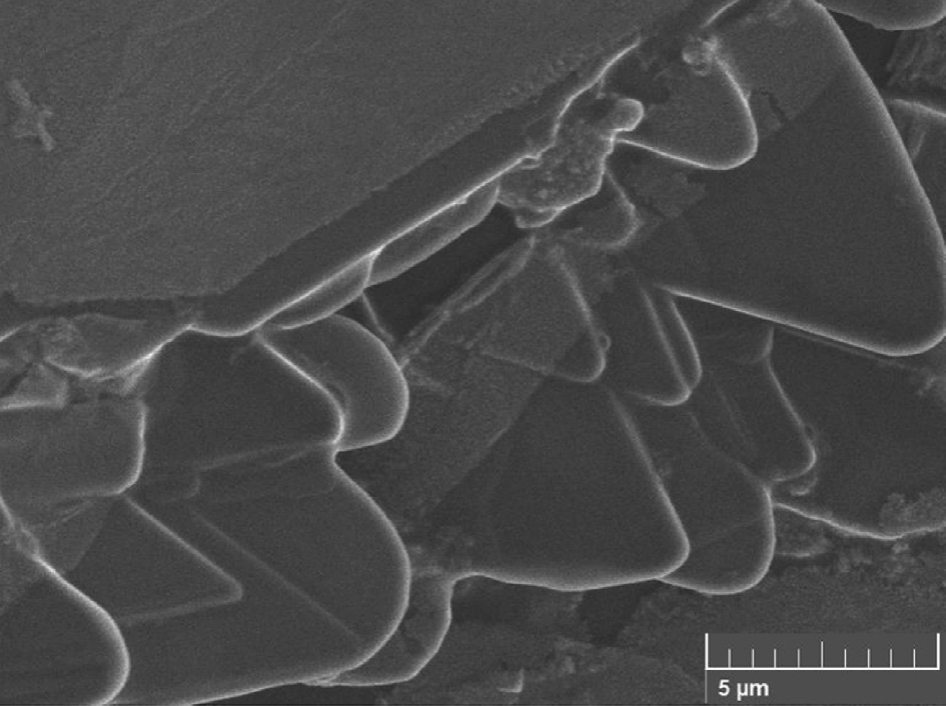
SEM image of Graphene.

**Fig. 6:**
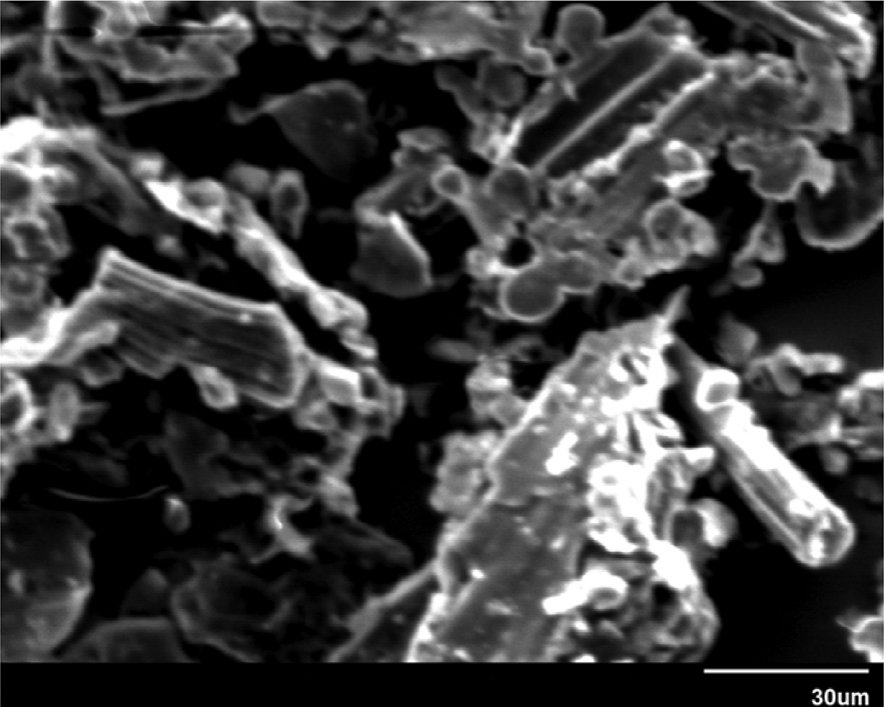
SEM image of Graphene Nanoscroll.

Fig. 5 shows a representative SEM image of as-produced GS, revealing the presence of thin stacked sheets, whereas Fig. 6 demonstrates that ultrasonication also resulted in the formation of long tubular nanoscrolls surrounded by few graphene layers.

### 3.3 Characterization of ND

**Fig. 7:**
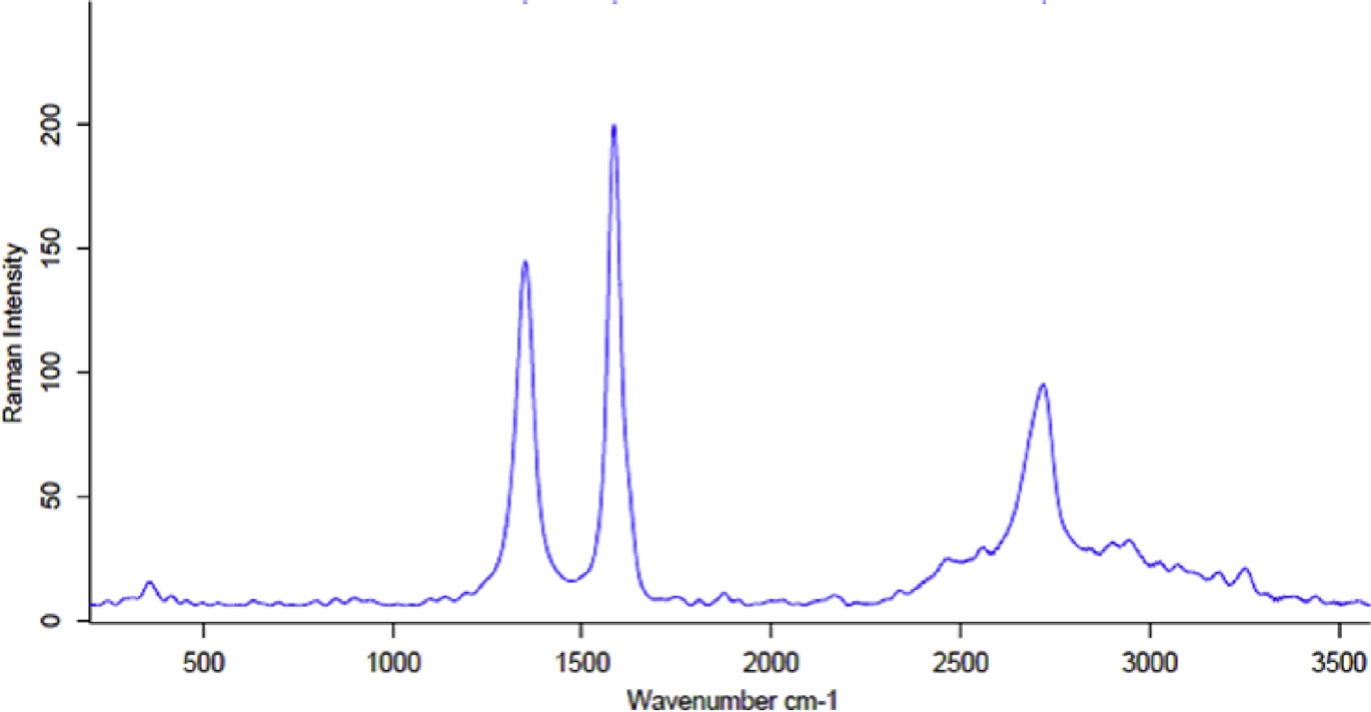
Raman spectrum of Nanodiamond.

Raman spectra of ND were dominated by the G bands of graphitic carbon and the strong ND line as observed in (Fig. 7). Notably, the small Raman scattering cross-section of diamond and the shielding effect of graphitic and amorphous carbon surrounding the diamond core resulted in D-band weakening. The broadening (full width at half maximum ˃50 cm^−1^) of the ND resonance and the appearance of a shoulder at ∼1260 cm^−1^ were ascribed to the small size of ND particles or/and the decreased size of coherent scattering domains separated by defects present in larger ND particles [20].

Another feature observed in the Raman spectra of as-prepared ND was the sharp symmetric peak at 1500–1800 cm^−1^, ascribed to the graphitic carbon band around 1584 cm^−1^ and its shoulder at ∼1750 cm^−1^ (attributed to the stretching of surface functional groups). The presence of graphitic carbon band around 1584 cm^−1^ refers to the presence of thin graphite layer (sp^2^ carbon atoms) surrounding diamond core. The second order overtone of the D band appears around 2700 cm^−1^ [21].

**Fig. 8:**
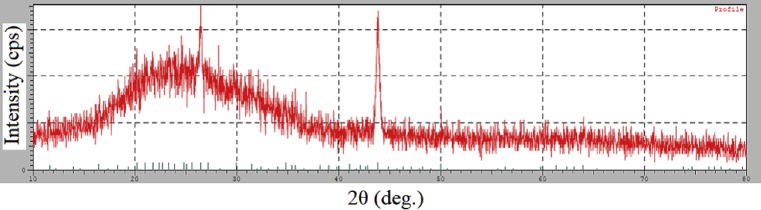
X- ray diffraction of nanodiamond.

X-ray diffraction (XRD) was used to determine the bulk structure by using a Cu Kalpha (1.544 Angstrom) monochromatic in (Fig. 8) exhibits abroad peak around 2θ = 20° that is attributed to the used quartz substrate. The sharp peak around 2θ = 26.4° is assigned to (002) graphite. Nanodiamond (111) is clear in the XRD spectra, located at 2θ 43.9° is in good agreement with previous studies [22].

**Fig. 9:**
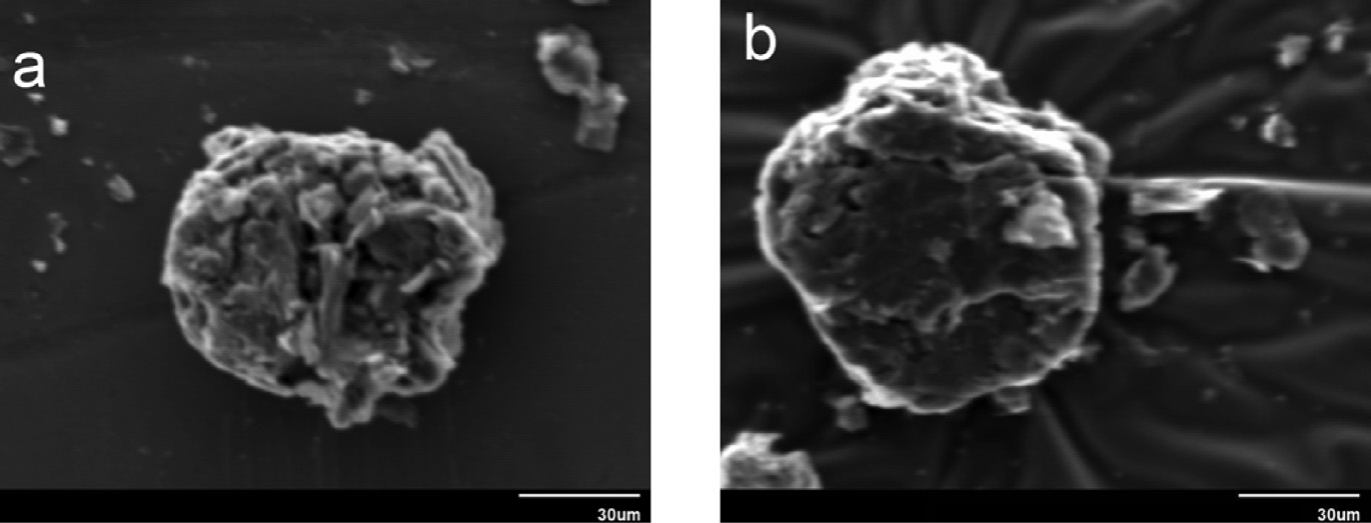
a, b): SEM images of the early stage of nanodiamond nucleation.

**Fig. 10:**
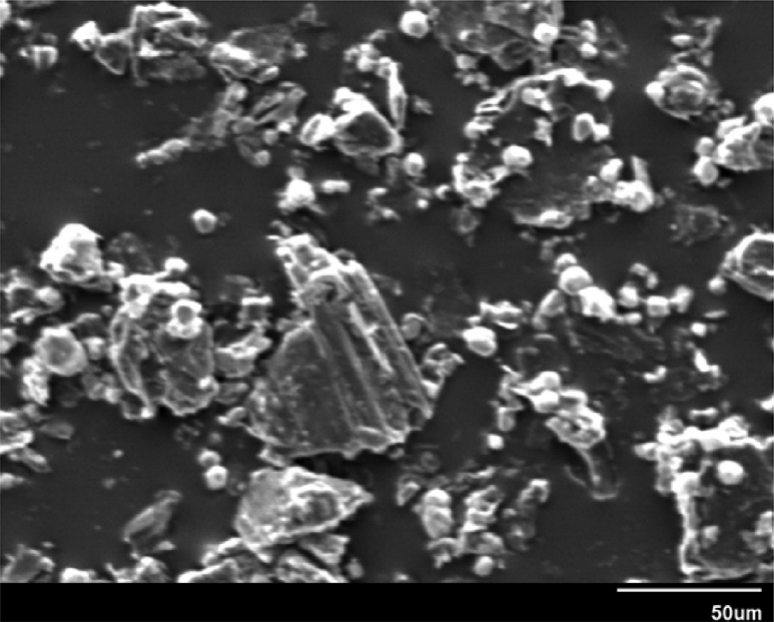
SEM image of Nanodiamond.

**Fig. 11:**
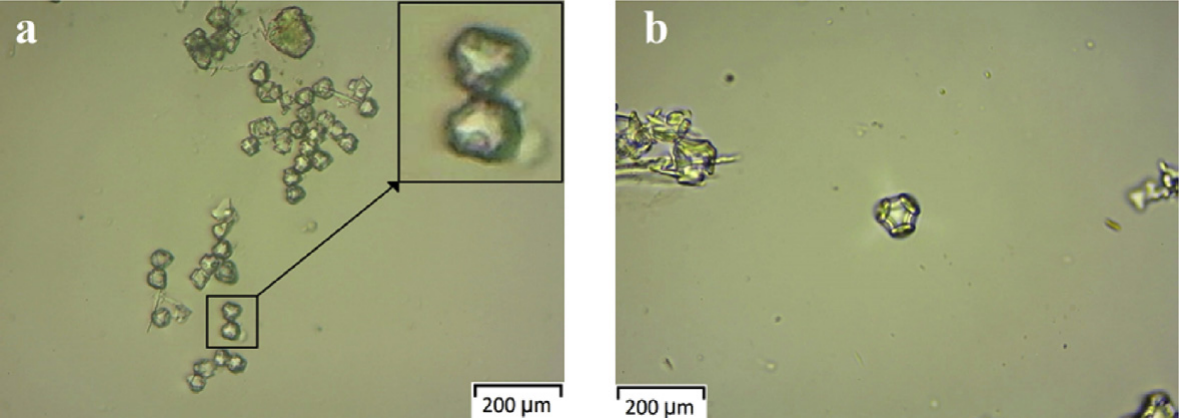
a, b): Micrographic image of the grown Nanodiamond.

Morphological and topographical characterization of ND was performed by high-resolution SEM imaging, with representative overview images shown in Figs. 9(a, b) and 10. Fig. 9 shows an SEM image of ND particle at an early growth stage, revealing that it exhibited rose-like morphology (sometimes termed “cauliflower” or “Ballas-type” [23] morphology) and tended to evolve into conventional diamond particles. Fig. 10 shows SEM image of as-prepared ND particles, some of which had a sparkled shape. Thus, some ND seeds had better growth chances than other ones. Fig. 11 (a, b) show those as-prepared ND particles which had been grown and exhibited facet-type morphology and reveals the presence of both single and twinned particles.

## 4 Conclusion

Herein, we prepared ND from graphite flakes via a cavitation-promoted process and demonstrated the role of cavitation medium composition, characterizing the morphology and structure of as-obtained ND particles by a range of instrumental analysis techniques. The simplicity, high yield, and upscaling potential of the developed method are expected to increase the application scope of ND and promote its industrial use.

## Declarations

### Author contribution statement

Haitham M. Al-Tamimi: Conceived and designed the experiments; Performed the experiments; Contributed reagents, materials, analysis tools or data.

Iman I. Jabbar: Conceived and designed the experiments; Performed the experiments; Analyzed and interpreted the data; Contributed reagents, materials, analysis tools or data.

Basma Al-Tamimi: Conceived and designed the experiments; Performed the experiments; Analyzed and interpreted the data; Contributed reagents, materials, analysis tools or data; Wrote the paper.

### Funding statement

This research did not receive any specific grant from funding agencies in the public, commercial, or not-for-profit sectors.

### Competing interest statement

The authors declare no conflict of interest.

### Additional information

No additional information is available for this paper.
